# Structure and Function of FS50, a salivary protein from the flea *Xenopsylla cheopis* that blocks the sodium channel Na_V_1.5

**DOI:** 10.1038/srep36574

**Published:** 2016-11-07

**Authors:** Xueqing Xu, Bei Zhang, Shilong Yang, Su An, José M. C. Ribeiro, John F. Andersen

**Affiliations:** 1 Guangdong Provincial Key Laboratory of New Drug Screening, School of Pharmaceutical Sciences, Southern Medical University, Guangzhou 510515, Guangdong, China; 2 The Laboratory of Malaria and Vector Research, NIAID, National Institutes of Health, Rockville, Maryland 20852 USA; 3 The Laboratory of Animal Models and Human Disease Mechanisms, Kunming Institute of Zoology, Chinese Academy of Sciences, Kunming 650223, Yunnan, China

## Abstract

Naturally occurring toxins have been invaluable tools for the study of structural and functional relationships of voltage-gated sodium channels (VGSC). Few studies have been made of potential channel-modulating substances from blood-feeding arthropods. He we describe the characterization FS50, a salivary protein from the flea, *Xenopsylla cheopis*, that exhibits an inhibitory activity against the Na_V_1.5 channel with an IC_50_ of 1.58 μM. The pore-blocking mechanism of this toxin is evident from the kinetics of activation and inactivation suggesting that FS50 does not interfere with the voltage sensor of Na_V_1.5. FS50 exhibits high specificity for Na_V_1.5, since 10 μM FS50 had no discernable effect on voltage-gated Na^+^, K^+^ and Ca^2+^ channels in rat dorsal root ganglia or VGSC forms individually expressed in HEK 293T cells. Furthermore, intravenous injection of FS50 into rats and monkeys elicited recovery from arrhythmia induced by BaCl_2_, as would be expected from a blockade of Na_V_1.5. The crystal structure of FS50 revealed a βαββ domain similar to that of scorpion β toxin and a small N-terminal βαβ domain. Site-directed mutagenesis experiments have implicated a basic surface including the side chains of Arg 6, His 11 and Lys 32 as potentially important in the FS50 Na_V_1.5 interaction.

The saliva of blood-feeding arthropods contains a rich mixture of pharmacologically active proteins that facilitate feeding and overcome the host’s defensive responses. Thus far, hundreds of distinct peptides and proteins that modulate the hemostatic, immune, and inflammatory responses of host animals have been purified and identified from hematophagous arthropod salivary glands^1^,^2^. Ion channels are pore-forming membrane proteins that play numerous crucial roles in the physiology of animals, including some that are potentially important for blood feeding. Many ion channel modulators and blockers have been obtained from animal venoms, such as those of spiders, scorpions, snakes, sea anemones, and sea snails. Only a handful of modulators are known from the salivary glands of hematophagous arthropods, however. Examples of these include vasotab from the saliva of the horse fly *Hybomitra bimaculata*, which prolongs the action potential and causes positive inotropism of isolated rat heart myocytes^3^ and Ra-KLP from the salivary gland of *Rhipicephalus appendiculatus* that activates maxiK channels in an *in-vitro* system^4^. Additionally, the salivary secretion of the blood-feeding insect *Triatoma infestans* has been shown to inhibit Na^+^ channels and decrease the generation and conduction of nerve action potentials, but the active constituents have not been isolated^5^.

Fleas are obligate blood-sucking ectoparasites of vertebrates, but little information is available on the function of individual substances in flea salivary glands. The FS family of peptides is unique to fleas and has been identified from the salivary glands of the rat flea *Xenopsylla cheopis (Rots)* and the cat flea *Ctenocephalides felis*^2^,^6^. FS proteins contain six to eight conserved cysteine residues forming three or four disulfide bonds and show relatively weak similarity to scorpion toxins. In this study, we describe the structure and function of a highly specific ion channel blocker from the salivary gland of *X. cheopis*, which belongs to the FS family and has been given the name FS50. We show that Na_V_1.5 is a target of FS50 in voltage clamp experiments using HEK 293T cells overexpressing this channel and show the specificity of the protein using dorsal root ganglion cells. We show that FS50 strongly suppresses the currents of Na_V_1.5 but not voltage-gated Na^+^, K^+^ and Ca^2+^ channels present on rat dorsal root cells. Additionally, we demonstrate that FS50 reduces the duration and frequency of arrhythmia in rats and monkeys induced by 2.0 mg/kg BaCl_2_, an activity consistent with the blockade of Na_V_1.5. Finally, we describe the three-dimensional structure of FS50 obtained by X-ray diffraction methods, and show that while it shares the classical core βαββ structure of scorpion β toxins, it does not appear to be a functional mimetic of these proteins. This work represents the first functional study of a protein from flea saliva, and will be helpful in understanding the blood-feeding mechanism of this species, in addition to providing a valuable probe of Na_V_1.5 channel.

## Results

### Effect of FS50 on the Na_v_1.5 channel

**Figure 1:**
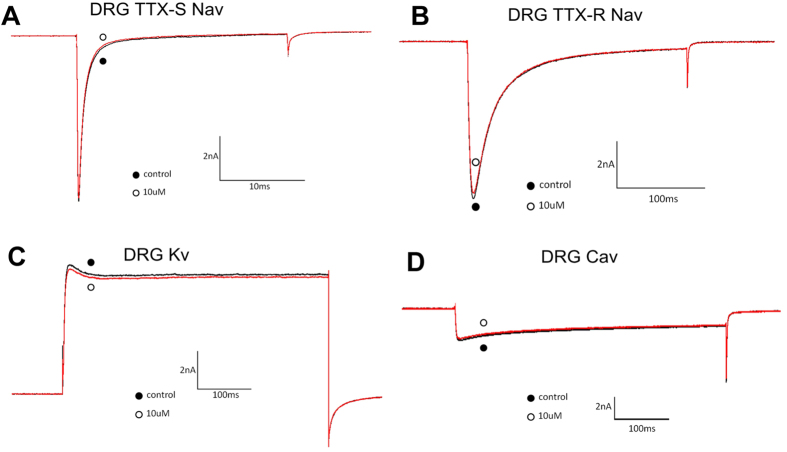
Effect of FS50 on Na+, Ca2+ and K+ channels on DRG. Sodium currents were evoked by a 50-ms step depolarization to −10 mV from a holding potential of −80 mV every 2 s. Potassium and calcium currents were evoked by a 500-ms step depolarization to 0 mV from a holding potential of −80 mV. By comparing traces recorded before (red) and after (black) application of 10 μM FS50, no discernable change was observed from TTX-S (**A**) and TTX-R (**B**) VGSCs, K+ (**C**) and Ca^2+^ (**D**).

**Figure 2:**
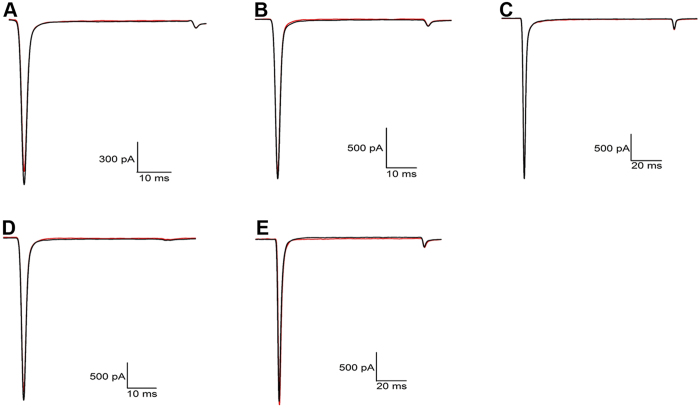
Effect of 10 μM FS50 on the activation of voltage gated sodium channels expressed in HEK293T cells. (**A**) NaV1.1, (**B**) NaV1.2, (**C**) NaV1.3, (**D**) NaV1.4, (**E**) NaV1.7. Black lines indicate currents in the absence of FS50 and red lines indicate activity in the presence of FS50.

**Figure 3:**
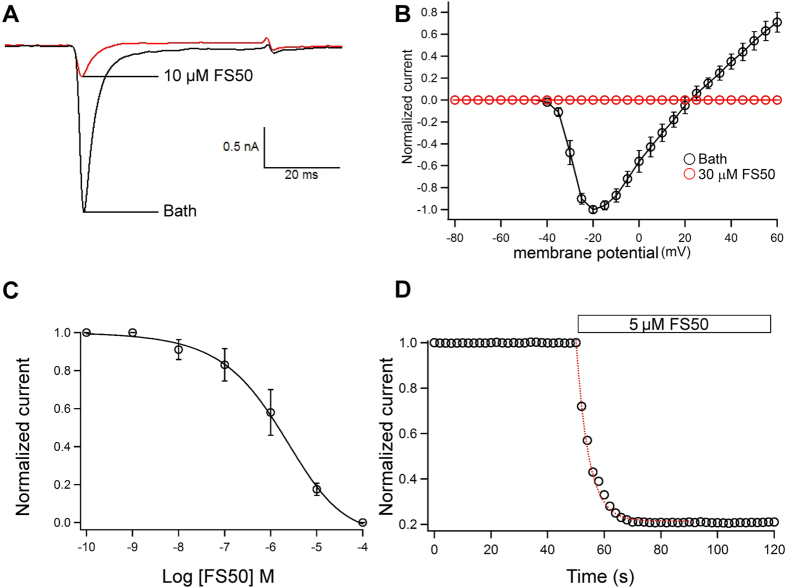
Effect of FS50 on Na_V_1.5 channels expressed in HEK 293T cells. (**A**) Representative Na_V_1.5 currents in the presence and absence of 10 μm FS50. (**B**) Relationship of the voltage-dependent Na_V_1.5 current to membrane potential in the absence of FS50 (filled circles) and in the presence (open circles) of 100 μm FS50. (**C**) The concentration-dependence of inhibition of Na_V_1.5 channels by FS50 (open circles). The solid line through the data is a fit to the Hill function. Each point represents the current (mean ± S.E. for 5 cells) relative to the control. (**D**) Inhibition kinetics of FS50 on Na_V_1.5 currents. The addition of 5 μM FS 50 in drug-free bathing solution is indicated by the bar.

Using the whole-cell patch clamp technique, the effect of FS50 was characterized on DRG neurons and HEK 293T cells expressing human Na_V_1.1, 1,2, 1,3, 1,4, 1,5 and 1.7. The experimental cells were held at a potential of −80 mV for at least four minutes to allow for adequate equilibration between the micropipette solution and the cell interior, and currents were then evoked using a 50-ms step depolarization to −10 mV repeated every second. As shown in Fig. 1A,B, 10 μM FS50 showed no evident effect on the normal activities of both TTX-S and TTX-R VGSCs in DRG neurons (*n* = 3). The currents of K^+^ and Ca^2+^ channels on rat DRG neurons were also completely insensitive to FS50 (Fig. 1C,D). In HEK 293T cells, FS50 at 10 μM concentration showed little or no effect on measured currents of Na_V_1.1, 1.2, 1.3, 1.4 and 1.7 when the membrane was held near its resting potential of −90 mV and currents were induced by a 50-ms depolarization to −30 mV (Fig. 2A–E). However, under the same conditions, the protein reduced the control peak amplitude of the Na_V_1.5 current 82.5 (±8.6%) (Fig. 3A). At a concentration of 30 μM, FS50 eliminated the remaining inward current completely (n = 4), and the inhibition of Na_V_1.5 currents was not overcome by depolarization to large positive test potentials (Fig. 3B). This irreversible inhibition is characteristic of a blocker that interacts with the pore region of Na_V_ channels^7^. To verify that toxin binding was concentration-dependent, the effect of FS50 was further measured on the human Na_V_1.5 subtype at concentrations ranging from 0.1 nM to 100 μM. The inhibitory effects of FS50 were concentration-dependent with an IC_50_ value of 1.58 ± 0.40 μM (Fig. 3C).

**Figure 4:**
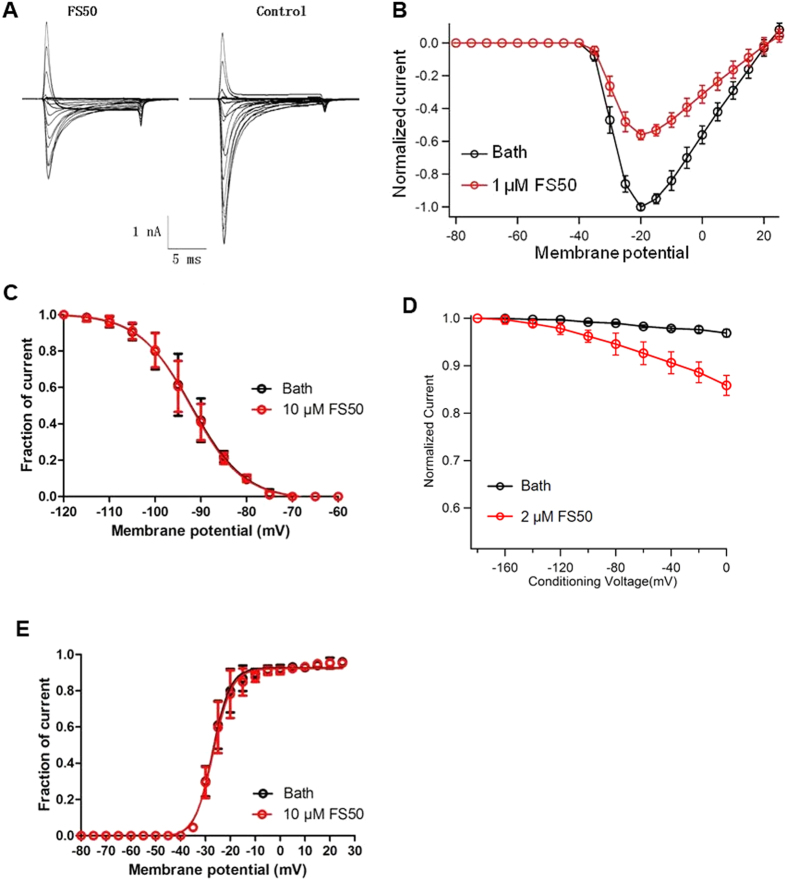
Effect of FS50 on the steady-state activation and inactivation of Na_V_1.5 channels. (**A**) A family of currents was elicited by 50 ms depolarizing steps to various potentials from a holding potential of −90 mV in the absence of FS50 (right) and after application of 1 μM FS50 (left). (**B**) The I-V curve of sodium currents showing the relationship between current traces before (black circles) and after (red circles) the addition of 1 μM FS50 in the experiment shown in A. In panel B, the data points represent the mean values obtained from four experimental cells (±S.E.). Test potentials ranged from −80 mV to +40 mV at increments of +5 mV. (**C**) Effect of 10 μM FS50 on steady-state fast inactivation of Na_V_1.5 channels. Currents were induced by 50 ms pre-pulses from −120 mV to −10 mV in steps of 5 mV, followed by a 50 ms test pulse to −10 mV. The data points (mean ± SE, n = 7) were fitted to the equation: I/I_max_ = 1/[1 + exp (V ± V_1/2_)/k]. Parameters obtained from fitting: V_1/2_ = −91.99 ± 0.17 mV for the control and −92.18 ± 0.21 for FS50-treated (n = 7). (**D**) The effect of 2 μM FS50 on steady-state slow inactivation. In the absence and presence of FS50, preparations were subjected to 10 s conditioning pulses from −170 to 0 mV, followed by a 100 ms pulse at −120 mV to allow recovery from fast inactivation, and then by a test pulse at −10 mV. (**E**) Steady-state activation kinetics estimated from current-voltage relationships in the absence or presence of FS50. Cells (n = 7) stably expressing Na_V_1.5 were stepped in 5-mV increments from −80 to +40 mV from a holding potential of −120 mV for 30 ms. For control cells and cells treated with 10 μM FS50, V_1/2_ = −27.15 ± 0.23 mV and −26.94 ± 0.29 mV, respectively.

To examine whether the depolarization of the membrane is a requirement for binding of FS50 to the Na_V_1.5 channel, the inhibition kinetics of FS50 were also characterized. At a concentration of 5 μM, FS50 produced a rapid inhibition with a time constant of 15 ± 2 s (Fig. 3D). The current-voltage relationship was determined using a 50 ms voltage step, ranging from −80 to +40 mV in steps of 5 mV from a holding potential of −90 mV at 2 s intervals. As can be seen from the current-voltage curve (Fig. 3B), currents were initially elicited at −40 ± 2.2 mV and the reversal potential was 20 ± 1.8 mV (*n* = 4). The peak of the current-voltage relationship was −20 mV for the control. After treatment with 1 μM FS50 for 1 min, the current amplitudes over the whole membrane potential range were reduced. There was not a significant shift of either the threshold, peak or equilibrium potential of I_Na_ under control conditions in the presence of FS50 (Fig. 4B), which implied that FS50 did not change the ion selectivity of Na_V_1.5.

To further investigate the mechanism underlying the inhibition, we characterized the effects of FS50 on steady-state inactivation and activation of Na_V_1.5. For measurement of steady-state fast inactivation, 50 ms pre-pulses from −120 mV to −10 mV in steps of 5 mV were used, followed by a 50-ms test pulse to −10 mV. FS50 at a concentration of 10 μM had no effect on fast inactivation (Fig. 4C), which was an effect similar to that of ProTx-II, a gating modifier toxin from the venom of the tarantula, *Thrixopelma pruriens*^8^,^9^. The effect of a sub-saturating (2.0 μM) concentration of FS50 on slow inactivation was also evaluated by subjecting cells to 10 s conditioning pulses from −170 to 0 mV, followed by a 100 ms pulse at −120 mV to allow recovery from fast inactivation, and then by a test pulse at −10 mV. The results show a significant negative shift in the voltage dependence of slow inactivation as a result of FS50 application (Fig. 4D). The conductance-voltage curves before and after the application of FS50 were plotted to further analyze the effect of FS50 on Na_V_1.5 activation. As illustrated in Fig. 4E, fits to the Boltzmann equation yielded completely superimposable curves showing a half-maximal activation voltage (V_1/2_) of −27.15 ± 0.23 mV in the control group and −26.94 ± 0.29 mV in the group exposed to 10 μM FS50. The effect of FS50 on the activation of Na_V_1.5 was different from those of classic β-scorpion toxins, which cause a hyperpolarized shift of VGSC activation^10^,^11^,^12^ and ProTx-II, which changes the voltage dependence of activation for Na_V_1.5 by producing an approximately 23 mV shift of the half-maximal activation voltage in the depolarizing direction when the cells are treated with toxin^13^,^14^. The fact that there were no changes in the slopes of the curves before and after the application of the toxin indicated that toxin binding might affect the cooperativity of the four voltage sensors of Na_V_1.5^15^. Taken together, these results suggest that the inhibitory action of the toxin is not voltage-dependent but acts through a voltage-independent pore-blocking mechanism.

### Assay of antiarrhythmia activity

**Figure 5:**
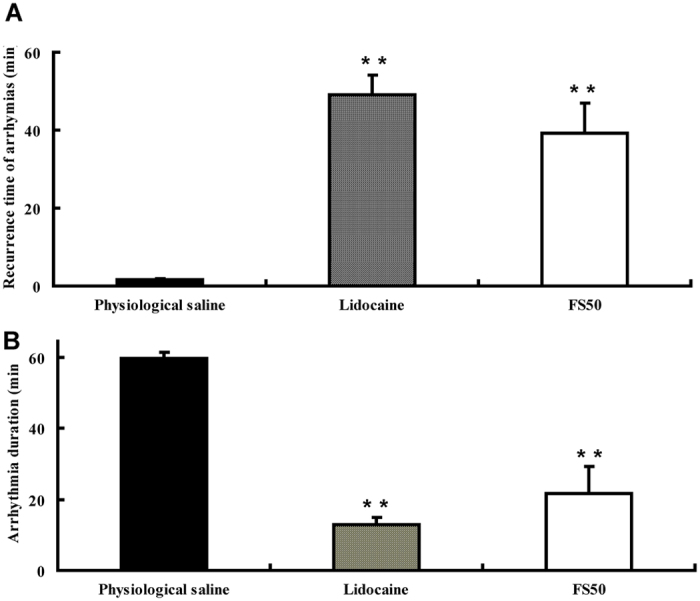
Inhibition of BaCl_2_-induced arrhythmia in rats by FS50. (**A**) Effect of FS50 (open bar) on the recurrence of arrhythmia in comparison to that of lidocaine (diagonally hatched bar) and a control of physiological saline (black bar). (**B**) The effect of FS50 on the duration of arrhythmia in comparison to lidocaine and the saline control. FS50 (0.5 mg/kg) was injected through venae sublingualis after induction of arrhythmia by 1 mg/kg BaCl_2_ (**p ≤ 0.01).

**Figure 6:**
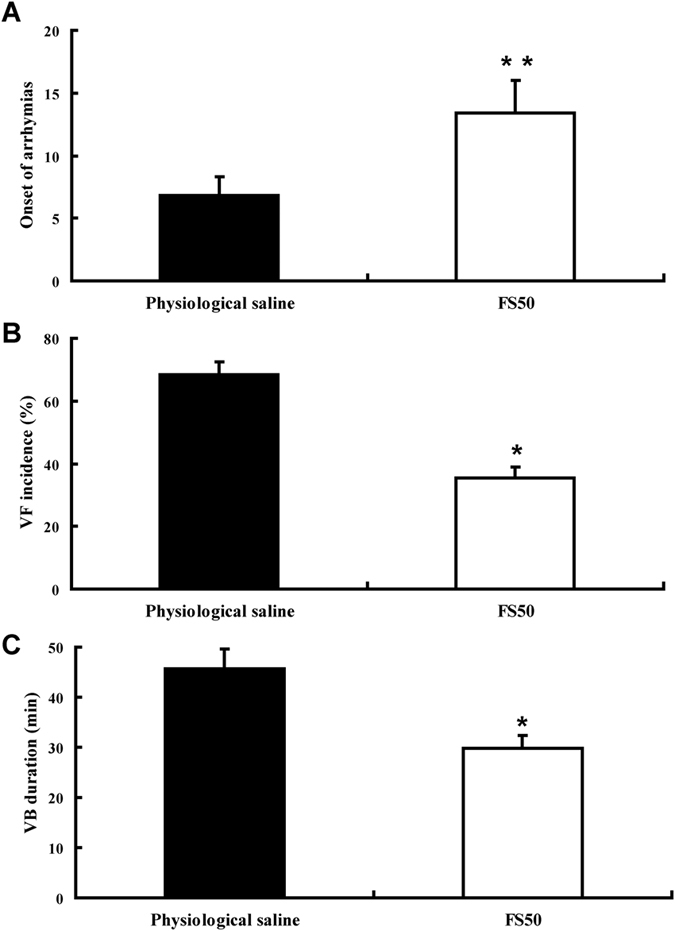
Inhibition of BaCl_2_-induced arrhythmia in macaques by FS50 . (A) Time (min) before the onset of arrhythmia after BaCl_2_ injection in animals treated with FS50 (2.5 mg/kg, open bar) or physiological saline (filled bar), **p ≤ 0.01). (**B**) Percentage incidence of ventricular fibrillation (VF) in animals injected with FS50 (open bar) or physiological saline (black bar, *p ≤ 0.05). (**C**) The duration of ventricular brachycardia (VB) induced by BaCl_2_, in the presence of FS50 (open bar) or physiological saline (black bar, *p ≤ 0.05)). In all experiments FS50 (0.4 mg/kg) was intravenously injected into the macaque 20 min before injecting 2.5 mg/kg BaCl_2_.

Figures 5 and 6 illustrate the effect of FS50 on arrhythmias induced by BaCl_2_ in rats and monkeys. Using the model of BaCl_2_-induced arrhythmia and compared with physiological saline, treatment with both FS50 and lidocaine significantly decreased the duration of arrhythmias and increased the recurrence time of arrhythmias in the rat (Fig. 5). Similarly, FS50 markedly reduced VB duration and VF incidence while delaying the onset of arrhythmias in the monkey (Fig. 6). These results indicate that FS50 is an anti-arrhythmia agent in rats and monkeys, an effect that would be consistent with blockade of Na_V_1.5 by the protein.

### The crystal structure of FS50

**Figure 7:**
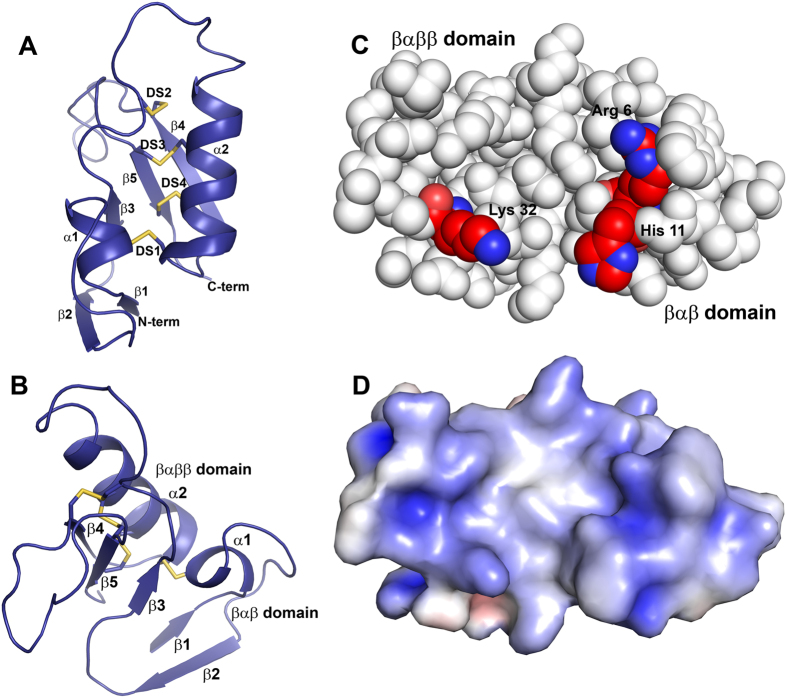
The crystal structure of FS50. (**A**) Ribbon diagram of the FS50 monomer showing the positions of α-helical (α1-α2) and β-strand (β−1-β5) structures. The positions of the four disulfide bonds are shown as sticks with sulfur being colored yellow, and labeled DS1-DS4. The N- and C-termini are also indicated. (**B**) A second view of FS50 shown as a ribbon diagram and giving the locations of the two domains labeled βαββ and βαβ. The α-helices and β-strands are also labeled and the disulfide bonds are shown as sticks. (**C**) Space filling model of FS50 showing the positions of the residues indicated as significant in mutagenesis studies. Arg 6, His 11 and Lys 32 are labeled and have carbon and oxygen colored in red and nitrogen in blue. (**D**) Electrostatic surface of FS15 from the same view as in panel C. The surface is contoured at ± 5 kT/e with blue indicating positively charged areas and red indicating negatively charged areas.

FS50 contains a cysteine-stabilized core βαββ structure having a general similarity to that found in the scorpion β toxins. The protein also contains a second domain made up of a small βαβ structure that is connected to the core domain through a coil region (Fig. 6A,B). While a second domain (the NC-globule) found in scorpion β-toxins is made up of elements both N- and C-terminal to the core domain, the βαβ domain of FS50 is comprised only of a long N-terminal extension. The C-terminus is truncated after β-strand C of the core domain and does not contribute to the second domain (Fig. 7A,B). The core-domain of FS50 is composed of a five-turn α-helix (residues 37–52) packed against a three-stranded antiparallel β-sheet (residues 25–27, 55–59, and 72–75) and is stabilized by three disulfide bonds (Cys 44-Cys 72, Cys 48-Cys 74 and Cys 31-Cys 59). In the second domain, the α-helix and two-stranded antiparallel β-sheet are formed by residues 5–9, residues 1–3, and residues 17–21, respectively. A fourth disulfide bridge between Cys 7 and Cys 52 connects the two domains. Notably, the loop connecting β-strands 1 and 2 of the core domain is elongated and folded back over the core domain β-sheet. Along with the βαβ domain, this arrangement produces a somewhat flattened surface unlike the protruding C-terminal sequence seen in the scorpion toxins. From its structure, it would appear that the binding mode of FS50 is unlike that of the scorpion toxins, and outside of general similarities in their overall folds, the two types of proteins probably function differently.

**Figure 8:**
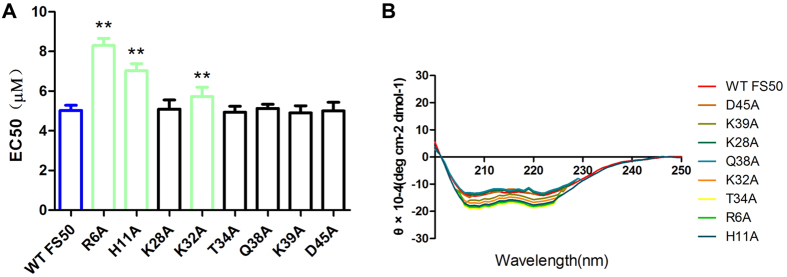
Site-directed mutagenesis of FS50 and its effect of blockade of Na_V_1.5. (**A**) Plot of the effectiveness of the wild-type and mutant variants of FS50 in the blockade of Na_V_1.5 currents in HEK293 cells. Bars represent the EC_50_ ± SD and asterisks indicate statistical significance (p ≤ 0.01, N = 5). (**B**) Circular dichroism spectra of samples of wild-type and mutant FS50 forms demonstrating that the mutant proteins do not show a significant difference from the wild-type in important secondary structural elements.

In order to identify sites of interaction between FS50 and Na_V_1.5, a panel of site-directed mutants was prepared, where a number of wild-type residues were changed to alanine. None of the eight mutations resulted in complete inactivation of the protein, but three mutations produced reductions in the effect of FS50 on the functioning of Na_V_1.5 when assayed in HEK 293T cells without disruption of the overall structure of the protein as indicated by analysis of circular dichroism (Fig. 8A,B). The three residues, Arg 6, His 11 and Lys 32, all lie in an approximate line running parallel to the large core α-helix and form a basic surface that may be a point of interaction with the channel (Fig. 7C,D).

## Discussion

Ion channel modulators could potentially serve a variety of functions in the salivary secretions of blood-feeding arthropods. Previous studies have suggested that blood-feeders prevent pain, itching and other sensations by limiting host inflammatory responses. Salivary scavengers of histamine, serotonin and cysteinyl leukotrienes are thought to delay the plasma extravasation and itching that accompany feeding^16^,^17^. Inhibitors of kinin generation and complement activation have also been described that act to lessen the pain of the bite and prevent swelling^18^,^19^. Inhibition of this sort would limit discomfort associated with inflammatory processes and prevent host behavioral responses that interfere with feeding. Ion channel modulators having analgesic effects compatible with these functions might be expected to occur in salivary secretions, but none have been identified as of yet.

Vasodilators are another important salivary component and several have been isolated from various species of blood feeders. While many of these target G protein coupled receptors^20^,^21^, only vasotab from the horse fly has been associated with both vasodilation and modulation of an ion channel^3^.

In this study we have shown that FS50, a salivary component of the rat flea, is a blocker of the sodium channel Na_V_1.5 that is capable of protecting rats and monkeys from barium chloride-induced arrhythmias. This channel is found in cardiac tissue, making it unlikely to play a significant role in flea feeding. However, toxins often show overlapping channel selectivity, and it may be that the natural target of FS50 has yet to be discovered. Examples of broadly selective toxins would include the centipede toxin μ-SLPTX-Ssm6a^22^, which inhibits the VGSCs Na_V_1.2 and Na_V_1.7, as well as the spider toxin JZTX-III^14^, which modulates the activity of sodium channels Na_V_1.4, and Na_V_1.5 and the potassium channel K_V_2.1. The relatively low potency of FS50 toward Na_V_1.5 may be indicative of the secondary nature of this channel as a target for the protein. However, the lack of effect on other individual Na_V_ forms or DRG neuron preparations suggests that the target is not another VGSC. Further screening may reveal that one or more peripherally significant channel forms is also affected by FS50.

The structure of FS50 is superficially similar to the scorpion toxins^23^. Both contain a conserved βαββ structure, but the molecular surface involved with channel interaction in the scorpion toxins is not recognizable in the flea protein^24^. Mutation of several residues, most notably Arg 6, to alanine reduces the potency of FS50, suggesting that a large basic surface of the protein contributes to is activity as a channel blocker. It does not appear that convergent evolution has resulted in a mimetic of the scorpion toxin, but rather, that the flea has produced a novel type of inhibitor from a similar scaffold. A diverse array of peptides related to FS50, but with varying sequence lengths, have been described in the transcriptome of the rat flea salivary gland, and we expect that peptides with additional receptor selectivity will be identified in future studies.

## Materials and Methods

### Ethics statement

All animal studies were carried out in accordance with guidelines set in experimental protocols approved by the Animal Care and Use Committee at Kunming Institute of Zoology, Chinese Academy of Sciences.

### Protein Expression and Purification

The cDNA for FS50 (genbank accession number ABM55452) was obtained from a previously described salivary gland library of *X. cheopis*^6^ and the position of the signal sequence cleavage site was predicted using the program Signal-P^25^. The cDNA sequence of FS50 was modified by PCR to remove the signal sequence, add a codon for the initiator methionine and insert NdeI and XhoI restriction sites used for cloning into the vector pET-17b. The expression plasmid was moved into the *Escherichia coli* cell line BL21(DE3)pLysS and expression was induced with 1 mM isopropyl β-D-thiogalactopyranoside at 37 °C. After an induction period of 3 h, the cells were harvested by centrifugation and washed with 20 mM Tris-HCl, pH 8.0. The cell pellet from 4 liters of culture was suspended in 100 mL of 20 mM Tris-HCl, pH 8.0, 0.2 mM phenylmethylsulfonyl fluoride, and the cells were lysed using a probe sonicator. The lysate was centrifuged at 30,000 × g and 10 °C for 30 min and the supernatant was dialyzed against 20 mM phosphate buffer, pH 8.0 for 24 h. After centrifugation and filtration, the proteins were purified by cation exchange chromatography on SP-Sepharose and size exclusion chromatography on Sephacryl S-100. The protein was further purified by additional gel filtration on Superdex 75. A selenomethionine derivative of FS50 was obtained by growing B834(DE3)pLysS with the plasmid construct described above in SelenoMet media supplemented with selenomethionine (Molecular Dimensions) as per the manufacturer’s instructions. The protein was purified using the methods described above.

### Patch clamp recording on rat dorsal root ganglion (DRG) neurons

Rat DRG neurons were acutely dissociated and maintained in a short-term primary culture according to procedures adapted from Xiao *et al*.^14^. Ca^2+^, Na^+^, and K^+^ currents were recorded from cells by means of the whole-cell patch clamp technique using an EPC-10 amplifier (HEKA, Lambrecht/ Pfalz, Germany). The P/4 protocol was used to subtract linear capacitive and leakage currents. Experimental data were acquired and analyzed by using Igor Pro.

### Transient transfection and patch-clamp recordings on HEK 293T cells expressing Nav1.5

Transient co-transfection of ion channel (WT) and green fluorescent protein constructs into human embryonic kidney (HEK) 293T cells were performed using the liposome precipitation method. HEK 293T cells were grown under standard tissue culture conditions (5% CO_2_ and 37 °C) in Dulbecco’s modified Eagle’s medium supplemented with 10% fetal bovine serum. Whole-cell patch-clamp recordings were carried out at room temperature (21 °C) using an EPC-10 amplifier (HEKA, Lambrecht/Pfalz, Germany). Fire-polished electrodes were fabricated from 1.7-mm capillary glass (VWR, West Chester, PA) using a P-97 puller (Sutter Instrument Company, Novato, CA). For whole-cell recording of HEK 293T cells overexpressing human Na_V_1.5, the standard pipette solution contained 140 CsF, 1 EGTA, 10 NaCl, and 10 HEPES (in mM), pH 7.3. The standard bathing solution was 140 NaCl, 3 KCl, 1 MgCl_2_, 1 CaCl_2_, and 10 HEPES (in mM), pH 7.3. After filling with pipette solution, the access resistance of electrode pipette ranged from 2.0 to 3.0 MΩ. The liquid junction potential for these solutions was <8 mV, and data were not corrected to account for this offset. The offset potential was zeroed before contacting the cell. After establishing the whole-cell recording configuration, the resting potential was held at −100 mV for 5 min to allow adequate equilibration between the micropipette solution and the cell interior. Linear leak subtraction, based on resistance estimates from four to five hyperpolarizing pulses applied before the depolarizing test potential, was used for all voltage-clamp recordings. Membrane currents were usually filtered at 5 kHz and sampled at 20 kHz. Voltage errors were minimized using 80% series resistance compensation, and the capacitance artifact was canceled using the computer-controlled circuitry of the patch-clamp amplifier.

### Antiarrhythmia effects of FS50 *in vivo*

Wistar rats weighing 200–220 g were placed into three randomly chosen groups and experiments were done one by one. Rats were anesthetized by intraperitoneal (i.p.) administration of 300 mg/kg chloral hydrate. The electrodes were injected subcutaneously into the tissue without contacting muscle and an electrocardiograph pattern for each rat was recorded for 60 min. Rats were monitored until the electrocardiograph pattern was stable for 3 min before beginning an injection of 1 mg/kg BaCl_2_ through venae sublingualis to induce arrhythmia. After arrhythmia was observed, 0.5 mg/kg FS50 was injected through the venae sublingualis. Control groups were injected with 0.9% isotonic saline or 15 mg/kg lidocaine instead of FS50. The recurrence time and duration of arrhythmias was noted and recorded. The effect of FS50 on BaCl_2_-induced arrhythmias was also measured in a primate model. Macaques were anesthetized with 300 mg/kg chloral hydrate and 0.4 mg/kg FS50 was intravenously injected 20 min before injecting 2.5 mg/kg BaCl_2_. A control group was injected with 0.9% isotonic saline instead of FS50 and all experiments repeated at least 3 times. The onset time of arrhythmia, the duration of ventricular bradycardia (VB) duration and the incidence of ventricular fibrillation (VF) was recorded and analyzed. Data are expressed as the mean ± SEM. Differences between the control and treatment groups were analyzed by the Dunnet t-test and paired t-test. Differences attaining a P-value < 0.05 were considered to be statistically significant.

### Crystallization and Data Collection

FS50 and its selenomethionine derivative were crystallized using the hanging drop vapor diffusion method in a solution consisting of 30% PEG 6000, 0.1 M bicine, pH 9.0. For data collection, the crystals were flash frozen in liquid nitrogen after a short soak in the crystallization buffer described above containing 10% glycerol. Data collection was performed at beamline 22-BM at the Southeast Regional Collaborative Access Team (SER-CAT), Advanced Photon Source (APS), Argonne National Laboratory. Native FS50 diffracted to 1.14 Å resolution in the space group C2 with two monomers in the asymmetric unit. The selenomethionine derivative of FS50 crystallized in the same form and diffracted to a resolution of 1.18 Å. Data were integrated and scaled using HKL2000 (Table 1)^26^.

### Structure Solution and Refinement

The structure of FS50 was determined using single anomalous diffraction (SAD) methods with data from crystals of a selenomethionine derivative of FS50 that contained only the N-terminal methionine residue introduced for expression in *E. coli*. (Table 1). Identification of a single selenium site derived from the initial (seleno)methionine residue of one of the molecules, and phasing were performed using SHELX C, D and E, as implemented in HKL2MAP^27^. Backbone traces containing parts of the two molecules in the asymmetric unit were built in an automated fashion using BUCCANEER^28^, and the remaining portions were built manually using Coot^29^. The model constructed using the selenomethionine data was then used to solve the structure of the wild type crystals using difference Fourier methods. Refinement of the wild-type structure was carried out using Phenix^30^. The coordinates and structure factor data for FS50 have been deposited in the RCSB Protein Data Bank with the accession code 5K6D.

## Additional Information

**How to cite this article**: Xu, X. *et al*. Structure and Function of FS50, a salivary protein from the flea *Xenopsylla cheopis* that blocks the sodium channel Na_V_1.5. *Sci. Rep.* **6**, 36574; doi: 10.1038/srep36574 (2016).

**Publisher’s note:** Springer Nature remains neutral with regard to jurisdictional claims in published maps and institutional affiliations.

## Figures and Tables

**Table 1:** Data collection, phasing and refinement statistics for FS50 and its selenomethionine derivative.

Crystal	Selenium	Wild-type
Resolution (Å)	22.3–1.18	22.0–1.14
Beamline	22-BM	22-BM
Wavelength (Å)	0.97471	0.96862
Completeness (total/high resolution shell)	99.9/84.0	98.6/92.0
Average Redundancy (total/high resolution shell)	3.0/2.2	3.6/2.7
R_merge_ (total/high resolution shell)	5.2/29.4	3.8/27.9
I/sigI (total/high resolution shell)	13.6/2.9	12.8/3.4
Observed reflections	274,336	185,913
Unique Reflections	46,796	51,086
Space group	C2	C2
Unit cell dimensions (Å)		
a	60.71	60.25
b	67.33	66.63
c	42.44	42.29
beta (˚)	122.26	121.69
Phasing statistics		
Number of selenium sites	1	
Contrast (SHELXE)	0.67	
RMS deviations		
bond lengths (Å)		0.006
bond angles (˚)		1.07
Ramachandran plot (Favored/Allowed)		99.4/100
Mean B value for all atoms		13.9
Coordinate error ML (Å, Phenix)		0.08
R_cryst/_R_free_		16.7/17.3
